# Differences in Physical Activity Recommendations, Levels of Physical Activity and Main Barriers to Exercise Between Spanish and Polish Cancer Patients

**DOI:** 10.3390/healthcare13060598

**Published:** 2025-03-09

**Authors:** Asier del Arco, Aitor Martinez Aguirre-Betolaza, Iker Muñoz Pérez, Ewa Malchrowicz-Mośko, Mateusz Krystian Grajek, Karolina Krupa-Kotara, Agata Wypych-Ślusarska, Piotr Nowaczyk, Tomasz Urbaniak, Arkaitz Castañeda-Babarro

**Affiliations:** 1 Department of Physical Activity and Sport Sciences, Faculty of Education and Sport, University of Deusto, 48007 Bilbo, Bizkaia, Spain; asier.delarco@opendeusto.es (A.d.A.); iker.munoz@deusto.es (I.M.P.); arkaitz.castaneda@deusto.es (A.C.-B.); 2 Department of Physical Activity and Sports Sciences, Faculty of Health Sciences, Euneiz University, Vitoria-Gasteiz, Alava, La Biosfera Ibilbidea, 6, 01013 Gasteiz, Araba, Spain; aitor.martinezdeaguirre@euneiz.com; 3 Department of Physical Education, Faculty of Tourism and Recreation, Józef Piłsudski University of Physical Education in Warsaw, Marymoncka 34, 00-968 Warsaw, Poland; ewa.malchrowicz-mosko@awf.edu.pl; 4 Department of Public Health, Faculty of Public Health, Silesian Medical University in Katowice, 40-055 Katowice, Poland; mgrajek@sum.edu.pl; 5 Department of Epidemiology, Faculty of Public Health, Silesian Medical University in Katowice, 40-055 Katowice, Poland; awypych@sum.edu.pl; 6 Breast Cancer Unit, Greater Poland Cancer Center, 61-866 Poznan, Poland; piotr.nowaczyk@wco.pl (P.N.); tomasz.urbaniak@wco.pl (T.U.)

**Keywords:** exercise, adherence, oncologist, neoplasm, lifestyle

## Abstract

**Background/Objectives**: Physical activity (PA) and exercise have demonstrated numerous benefits for patients with cancer. However, there may be different barriers which vary according to geographical area. The aim of this study was to compare oncologists, PA recommendations, PA patterns and barriers in two different geographical areas. **Methods:** A total of 254 patients were included, 239 of them women and 15 of them men, while 41.3% of the sample was Polish and 58.7% of the sample Spanish. **Results:** In terms of differences in time spent on PA per day, the Spanish spent more time walking than the Polish did (*p* = 0.007). However, no significant differences were found between countries, types or intensity of exercise, and there was a significant relationship between age and type of PA modality (*p* = 0.002). At the same time, there were different reasons for not practicing PA (*p* = 0.009). The subsequent analysis showed that younger adults were prone to more vigorous-intensity exercise than the other age groups (*p* = 0.001, η^2^ = 0.08). Furthermore, there was a significant difference between age groups, countries and sitting time (*p* = 0.01), with Polish patients spending more time sitting than Spaniards (*p* = 0.01, η^2^ = 0.06). **Conclusions:** Although PA patterns in the two countries were similar, the main barriers to exercise differed. Therefore, PA programs should be as personalized as possible (taking into account sociodemographic, cultural and climatic characteristics). Ultimately, to improve the quality of life and health of their patients, oncologists should provide those activities that are most beneficial to their patients.

## 1. Introduction

Exercise and (PA) have demonstrated numerous health benefits for the treatment or improvement of different pathologies, among which we find cardiovascular diseases, metabolic disorders and multiple sclerosis. Cancer is another pathology that can benefit from exercise and PA. Among these benefits, we can find an improvement in anxiety, depressive symptoms, fatigue, physical function and health-related quality of life [1]. However, when it comes to implementing physical exercise programs, problems exist with adherence levels, with these being understood as referring to the extent to which an individual’s conduct corresponds to the agreed plan of the exercise intervention suggested [2]. With regard to physical exercise programs in cancer patients, low levels of adherence have been reported [3,4].

There are many barriers attached to the system, physicians and patients themselves in terms of the practice of PA [5]. In the case of patients, some of the main barriers are physical-related issues, time pressure and low motivation or interest [6]. There is another major barrier with regard to oncologists or people working in clinical settings, due to their lack of clarity when advising or referring patients to do exercise [7]. This reinforces the fear that may exist towards PA or the lack of knowledge regarding the benefits of exercise programs. As for those oncologists who recommend PA, we can observe that less than half of them do so (46%) [8]. In the same way, there may be differences between what they recommend and what is perceived by patients. Thus, it is important to assess the information received by the patient in order to ascertain the effect this information has on adherence (or compliance with recommendations) to PA.

One variable that can greatly condition the extent to which PA is undertaken, as well as the barriers to doing so, is the geographical location. The area or country where patients live can influence their PA patterns in two main ways: firstly, in a social and cultural way and secondly in an environmental way [6]. Of the different barriers perceived by patients, one of them is the climate [9], which can lead to the need for space and materials [10]—another barrier to the realization of physical exercise programs. On the other hand, different studies have highlighted the importance of receiving recommendations from oncologists [11] or receiving feedback from them regarding exercise intervention [12] as key factors in determining adherence to exercise. Although oncologists’ recommendations should be adapted to the characteristics of each area of residence [climatic, social and cultural], to our knowledge, no work thus far has studied or compared the recommendations received by cancer patients in different geographical areas.

The aim of this study is therefore to find out what PA recommendations patients say their doctors have suggested to them. On the other hand, it aims to determine the amount of PA undertaken in order to assess the influence of these recommendations on the type and amount of PA carried out (adherence to or in compliance with the oncologist’s recommendations). Finally, the differences between recommended and undertaken PA will be compared between two different geographical areas (Spain and Poland).

## 2. Methods

### 2.1. Study Design

A descriptive, cross-sectional research study was conducted, and an online questionnaire with a link to Google Forms for the patients to fill in was distributed among different Spanish and Polish patients in order to carry out research into their PA patterns. There were two main requirements for participation in the study: firstly, to be diagnosed with cancer and be undergoing treatment at the time the research was carried out, and secondly, to be over 18 and 21 years of age (in Spain and Poland, respectively).

### 2.2. Instruments

The questionnaire was designed by researchers at the University of Deusto, written in Spanish, and then literally translated into Polish, combining multiple choice and scale or open response options. The questionnaire contained the short Spanish [13] and Polish [14] versions of the International Physical Activity Questionnaires (IPAQ), respectively, for each country. In addition to the questions collected by the IPAQ, personal data (i.e., age, gender, etc.) and sociodemographic information (place of residence) was collected to gather the subjects into their age group and make comparisons between the two populations (Spain and Poland). The aim of the last part of the questionnaire was to research the reasons for not engaging in PA, whether their oncologists had recommended PA and, if so, what activity had been recommended. The data collection took place between January and February 2023.

### 2.3. Procedures

The researchers sent the questionnaire online via social networks to the oncologists, who then forwarded the survey to their patients during their check-ups. Those patients who met the inclusion criteria were informed by their oncologists about the aim of the study before they started to fill in the questionnaire and that by filling in the survey, they were giving their consent to participate. Additionally, they received contact information about the study directors in case they had any doubts or wished to terminate their participation at any time. The study was conducted according to the guidelines set out by the Declaration of Helsinki and approved by the Ethics Committee at the University of Deusto (PI2023087, approved 12 July 2023).

### 2.4. Statistical Analysis

Data homogeneity of variance was performed ascertained using Levene’s test, and the Kolmogorov–Smirnov, Cramér–von Mises and Anderson–Darling tests were used to analyze the normal distribution of all continuous variables. The variables registered were expressed using frequency tables, while Student T-tests (or non-parametric homologous tests) were used to compare walking time, moderate and vigorous PA time and sitting time per week between countries (Spain vs. Poland). If any significant differences were found, Cohen’s d was used to measure the effect size [ES], employing the small (*d* = 0.2), medium (*d* = 0.5) and large (*d* = 0.8) reference values to interpret them, as suggested by Cohen [15]. If a non-parametric test was used (Mann–Whitney U test), a Rank-Biserial Correlation test was used in order to establish ES (ranging from −1 to 1).

Pearson’s χ^2^ test was used to examine any associations between categorical variables (age group, type of PA recommended by the oncologist, reasons for not undertaking PA and nationality). To determine whether previous analyses showed any significant associations, the Phi coefficient or Cramér’s V was used to establish the magnitude of association. Subsequently, correspondence analysis (CA) was used to demonstrate the relationship between any categorical variables that had previously shown an association.

Four multiple regression models were set with walking time, moderate and vigorous PA time and sitting time per week as the dependent variables, and age group, nationality and the interaction age group × nationality as possible predictors. A stepwise regression approach based on Ordinary Least Squares (OLS) was provided with the aim of determining the best explanatory independent variables, with the R package olsrr (version 0.6.1) being used for this analysis [16].

Additionally, the R package dplyr (version 1.1.4) [17] was used to identify possible outliers and to improve the fitting of the regression model. Outliers were identified and removed in the multiple regression model when the absolute value of the studentized residual (SRE) was ≥2, while model performance was assessed using the root mean square error (RMSE) and Pearson’s R^2^.

The Jarque–Bera test was used to check the normal distribution of residuals in the regression models, and the homoscedasticity of these models was ascertained using a χ^2^ test.

If any of these regression models showed significance, an ANOVA test (or its non-parametric counterpart) and the relevant post hoc Holm–Bonferroni test were conducted in order to establish possible significant differences between the groups. To define the magnitude of any significant difference in ANOVA, partial eta squared (η^2^) was used to measure ES, employing the small (η^2^ = 0.01), medium (η^2^ = 0.06) and large (η^2^ = 0.14) reference values. If the test shown was non-parametric, epsilon (ε^2^) was used as the ES (ranging from 0 to 1).

R software 4.2.2 (Vienna, Austria; 2022) and RStudio (version 2022.12.0.353; Boston, MA, USA; 2022) were used for the statistical analyses, and the significance level was established as *p* ≤ 0.05.

## 3. Results

A total of 254 patients participated in the present study, of whom 94.09% were women (n = 239) and 5.91% were men (n = 15). Regarding nationality, 58.4% (54.95% women, 3.45% men) of the sample was Spanish, and 41.6% was Polish (all women). Regarding age group, 5.9% (n = 15) were <30, 6.7% (n = 17) between 30 and 40, 30.3% (n = 77) 41–50, 34.3% (n = 87) 51–60, 19.7% (19.7%) 61–70 and 3.1% (n = 8) 71–80 years old. Table 1 shows the sample’s principal descriptive characteristics.

Following outlier analysis, a final sample of 227 patients (204 women and 23 men) was taken into consideration for the subsequent statistical tests.

With regard to possible significant differences based on nationality between PA, intensity (vigorous and moderate), time of practice and time per day walking were taken into account. The analysis (Table 2) only showed a significantly longer walking time for Spaniards compared to Poles. In this respect, Spaniards were more likely to engage in low-intensity physical activity than Poles. However, due to the low ES detected, these results should be interpreted with caution. As shown in Table 2, no additional significant differences were found in the time spent on each activity based on participants’ nationality, suggesting homogeneous lifestyle habits within this population.

**Figure 1:**
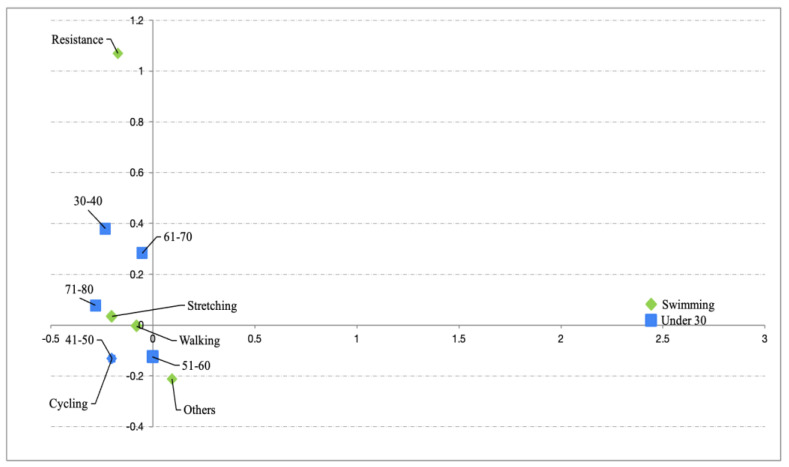
Relationship between PA recommended and age group.

**Figure 2:**
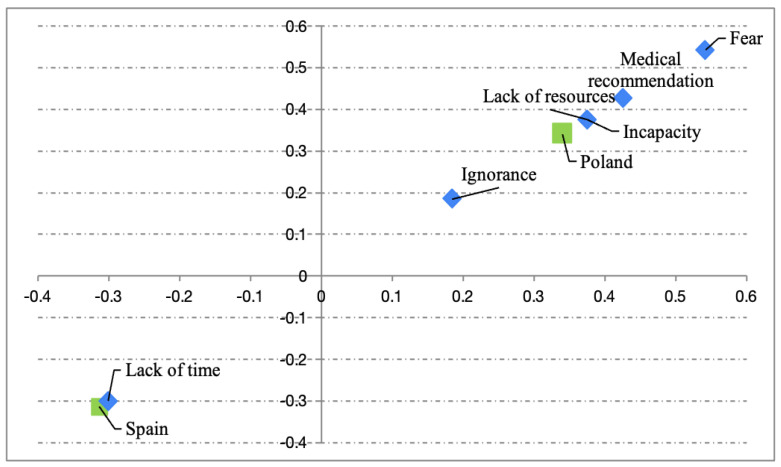
Relationship between the reason for not engaging in PA and nationality.

Several Pearson’s χ^2^ tests were conducted between categorical variables (i.e., age group, type of PA recommended by oncologists, reasons for not undertaking PA and nationality), which only showed a significant relationship between the type of PA recommended and age group (χ^2^ (25) = 49.8, *p* = 0.002; Cramér’s V = 0.25) and reasons for not undertaking PA vs. nationality (χ^2^ (5) = 15.4, *p* = 0.009; Cramér’s V = 0.3). Subsequent CAs suggested (Figure 1) an association of swimming as the most recommended PA for patients under 30 years of age and cycling as the most recommended among the 31–40 age group. The second CA test revealed a significant reason for not undertaking PA based on nationality (Figure 2). In this sense, the main reason given by the Spanish was lack of time, while the Poles gave inability and lack of resources as the main reasons for non-practice of PA.

In the regression models, the first model (with walking time as the dependent variable) showed that only nationality explained 10.1% of the variance (Adj-R^2^ = 0.06, *p* = 0.007; RSE = 32.5 min). The second regression model did not show any significant predictive capacity (*p* = 0.83), while the third OLS stepwise regression found that only the age group could significantly explain vigorous PA time (Adj-R^2^ = 0.07, *p* = 0.001; RSE = 36.79 min). The fourth OLS regression model (with sitting time as the dependent variable) showed that the independent variables of age group, country and their interaction were capable of explaining variance in the dependent variable (Adj-R^2^ = 0.05, *p* = 0.02; RSE = 2.89 min).

**Figure 3:**
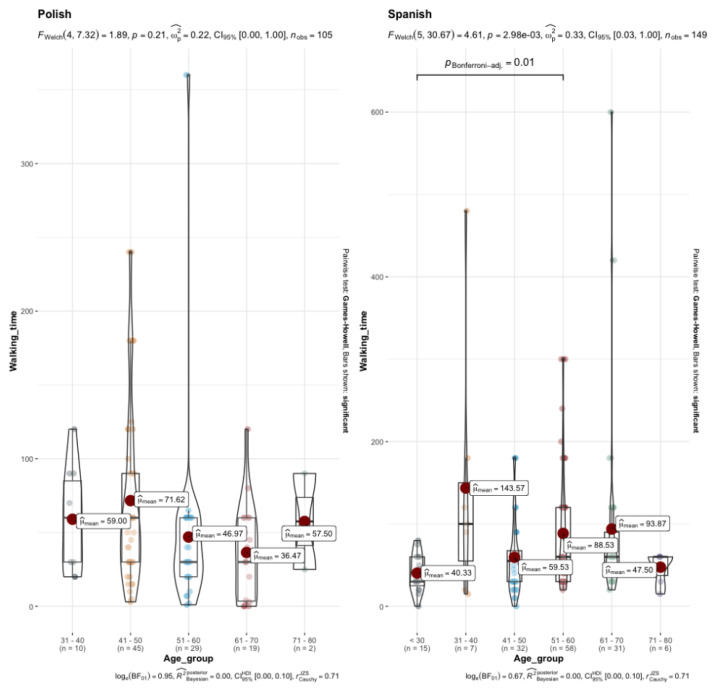
Differences in walking time based on age group and nationality. Note: exponent notation is shown as ‘e-X’.

The first ANOVA (age group × time of vigorous PA intensity) showed significant differences (*p* < 0.001, ε^2^ = 0.08), while the post hoc test showed significant differences between the <30-year-old age group and 51–60 group in terms of walking time (Figure 3). Although the differences were not statistically significant, it is worth noting that the age groups with the shortest walking time varied by nationality. Among Polish participants, this was the 61–70 age group, whereas for Spanish participants, it was those under 30.

**Figure 4:**
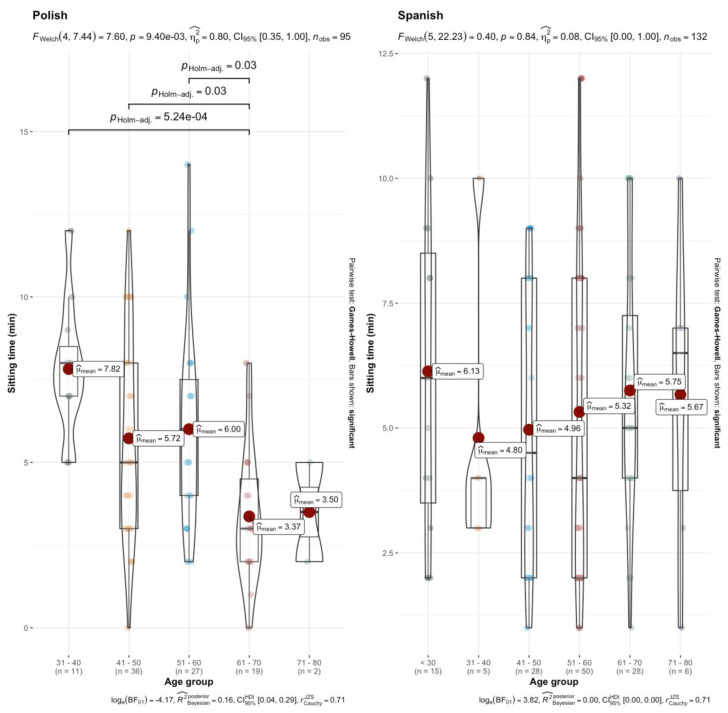
Differences in sitting time based on age group and nationality. Note: exponent notation is shown as ‘e-X’.

The second ANOVA test (age group × country × sitting time) showed significant differences (*p* = 0.01, η^2^ = 0.06), while post hoc analysis revealed significant differences among four age groups, albeit only in Poles (Figure 4). Spanish participants did not show any significant differences between age groups in terms of sitting time (*p* = 0.77). For this reason, and for the purpose of better understanding, two non-parametric one-factor ANOVAs were conducted, split by nationality (Figure 4). Although not statistically significant, this analysis suggests a trend in the Polish population toward less sitting time with increasing age. Conversely, among Spaniards, sitting time tends to increase with age, except for the oldest age group in both populations.

## 4. Discussion

The objective of this research was to find out what PA recommendations patients say their physicians have recommended to them. On the other hand, it also aimed to analyze whether there were differences in the PA undertaken and the main barriers to doing so among Spanish and Polish patients. The results indicate that there are small differences in PA between cancer patients of both countries, whereas barriers also seem to differ between these geographical locations.

With regard to the time spent on each type of PA (moderate or vigorous), as well as sitting/walking time, differences were only observed in walking time, these being higher in Spaniards and with a small effect size. As for determinants that might influence post-diagnosis PA, several factors such as younger age [18], a higher education level [19] or higher pre-diagnosis PA [20] were identified, which could influence both the type and time of PA undertaken. On the other hand, when analyzing the main barriers to not doing PA in Polish patients, we observed that disability and lack of resources were the two main ones. In understanding the pursuit of PA as a multifactorial phenomenon and analyzing patients’ responses, some patients argued that weather conditions (i.e., darkness too early, rain or cold) were the main barrier to not being physically active, which has been previously stated [21]. In addition, the lack of men among the Polish patients may also have affected the results, since women are less likely (about 30%) to adhere to PA recommendations among cancer survivors [22]. However, in addition to cultural differences [23], it is important to highlight structural barriers, such as the lack of rehabilitation programs or the lack of financial incentives from countries [24], despite evidence supporting their implementation.

The Spanish patients argued that lack of time was their main reason for not engaging in PA, which could be affected by both work and leisure time. In this sense, the work environment (time at work, type of work, unemployment or job strain, etc.) seems to condition PA patterns [25]. Likewise, it is important to differentiate between leisure PA and occupational PA, as these may not offer the same health benefits [26].

Regarding the PA recommended by oncologists, only two activities were highlighted in different age groups. Firstly, for those under 30, swimming was recommended to a greater extent, while for the 31–40 age group, cycling was the most recommended activity. The benefits of predominantly oxidative activities in cancer patients are well known [27], to the extent that many official recommendations focus on these activities rather than on resistance work. However, some of the therapies applied with cancer patients, such as androgen deprivation therapy, can compromise bone health by decreasing bone mineral density [28]. This can lead to an increased risk of hospitalization as well as a higher likelihood of fractures [29]. Therefore, it is important that the activities recommended by oncologists do not focus solely on sports such as swimming or cycling and include activities with impact such as running, or resistance training, which have been shown to improve bone mineral density [30]. In this regard, some official guidelines such as those provided by the American College of Sports Medicine or the International Multidisciplinary Round Table Consensus from 2019 recommend two days per week of muscle resistance activities for each major muscle group [1,31]. Therefore, resistance training is crucial in cancer patients, due to the levels of cachexia some patients evidence and the effect this activity has on increasing muscle mass, with major clinical implications [32].

With regard to the prediction models, the first shows how walking time is partly explained by nationality (10.1%). As mentioned above, there are numerous factors that condition the pursuit of PA and barriers differ between nationalities. Nationality was the only explanatory factor and, although the rank-biserial correlation coefficient was not extremely high, it did have a role in conditioning walking time. However, when referring to the responses of the subjects, there are very different reasons that limit PA or walking time, among which we find lack of time, being overweight, laziness, fatigue associated with cancer, lack of interest, perception of it as a boring activity or prioritizing work and social life. This heterogeneity shows how PA programs should be of a personalized nature, adjusted to the characteristics of each subject.

In the second model, with vigorous PA as the dependent variable, the age group was found to significantly condition the time spent on this modality. In terms of the differences between age groups regarding different activities, there were differences in the time taken in vigorous PA between the group < 30 years and the groups comprising 51–60 and 61–70 years of age. This shows a trend towards less vigorous PA as age increases. One of the best indicators of mortality and life expectancy in both cancer patients and the general population is cardiorespiratory fitness [33]. In this regard, high-intensity interval training (HIIT) (which would be classified as vigorous training) has been shown to improve cardiorespiratory fitness and body composition to a greater extent than moderate training [34,35], with training programs of shorter duration. On the other hand, in colorectal cancer patients, a HIIT training program was more effective than a moderate-intensity program in improving maximal oxygen uptake and muscle mass and decreasing fat mass [36]. Therefore, although these programs may be more demanding due to increased intensity, it is important that they are undertaken by patients regardless of their age.

The last model, which aimed to analyze which variables conditioned sitting time, showed that age group, country and their interaction explained the dependent variable. When analyzing the differences between age groups for sitting time, differences were only observed in Polish patients, not in Spanish ones. Sitting time was less as age increases, whereas in Spanish patients there was no clear trend as the patient’s age increased. This difference could derive from the time spent at home and the climate in Poland, which has shown to condition the PA patterns in cancer patients [21]. Sitting time, which in cancer patients can be up to more than 4 h, is related to free fat mass and fatigue [37]. This sedentary behavior also appears to increase some inflammatory biomarkers. Thus, exchanging sitting time for 10 min of PA is associated with less fatigue and a higher quality of life [38]. In this sense, it is important that oncologists emphasize that patients should remain physically active and ascertain whether they adhere to or exceed official PA recommendations.

Although our work offers new findings, it has a number of limitations. Firstly, we only analyzed patients from two countries that were part of the same continent, so future research should consider other countries and preferably from more continents. Also, among the Polish patients, there were no responses from men, which might have conditioned the results. Finally, there may be a possible source of bias in the final two questions of the questionnaire about physical activity recommendations received by patients from their oncologists.

## 5. Conclusions

In conclusion, PA patterns seem to differ according to the patient’s country; strategies to promote PA should therefore be carried out taking into account the sociodemographic, cultural, climatic and structural characteristics, in an attempt to make them as personalized as possible. Finally, it is important for oncologists to be aware of the activities that can be of most benefit to patients and to insist on limiting sedentary behaviors.

## Figures and Tables

**Table 1:** Descriptive PA habits of the sample based on nationality and gender.

				Frequency of Vigorous PA (Days per Week)		Frequency of Moderate PA (Days per Week)		Frequency of Almost 10 Min Walking (Days per Week)	
Nationality	Gender	Age Group	n (Relative %)	Mean	SD	Mean	SD	Mean	SD
Spanish	Women	<30	7 (2.8%)	1.71	0.95	2.14	2.41	5.86	1.95
		30–40	7 (2.8%)	2.00	1.73	2.43	2.64	6.71	0.756
		41–50	30 (11.8%)	2.50	1.89	2.13	1.83	5.53	1.93
		51–60	54 (21.3%)	1.61	1.99	2.44	2.27	6.20	1.50
		61–70	26 (10.2%)	1.27	1.95	2.00	2.30	6.58	1.10
		71–80	2 (0.8%)	0.500	0.707	4.50	3.54	4.50	3.54
	Men	<30	8 (3.1%)	4.50	1.93	2.50	2.45	5.50	2.62
		30–40	0	-	-	-	-	-	-
		41–50	2 (0.8%)	1.50	2.12	2.50	3.54	7.00	0.00
		51–60	4 (1.6%)	1.75	2.06	2.25	2.87	2.75	3.10
		61–70	5 (2%)	0.600	1.34	1.60	1.52	4.80	2.39
		71–80	4 (1.6%)	2.25	1.71	3.50	3.51	5.75	1.50
Polish	Women	<30	0	-	-	-	-	-	-
		30–40	10 (3.9%)	2.00	1.76	2.10	1.60	6.30	1.57
		41–50	45 (17.7%)	1.11	1.48	2.49	2.07	6.00	1.57
		51–60	29 (11.4%)	2.28	2.53	3.28	2.85	6.00	1.60
		61–70	19 (7.5%)	1.89	1.97	2.53	1.95	5.00	2.83
		71–80	2 (0.8%)	2.00	0.00	4.00	2.83	6.00	1.41

Data are shown as absolute and relative frequencies. SD: Standard deviation. No data are provided for Polish men due to non-response.

**Table 2:** Time differences in PA intensity, walking and sitting time between nationalities.

	Nationality	N	Mean	SD	*p*	Rank-Biserial Correlation
Time spent on vigorous PA (min per day)	Spanish	132	33.63	40.67	0.42	0.06
	Polish	95	27.71	34.03		
Time spent on moderate PA (min per day)	Spanish	132	35.98	49.85	0.51	0.05
	Polish	95	35.72	44.05		
Walking time (min per day)	Spanish	132	57.46	34.67	0.007	0.21
	Polish	95	44.40	30.61		
Sitting time (hours per day)	Spanish	132	5.42	3.00	0.721	0.03
	Polish	95	5.53	2.93		

SD: Standard deviation; *p*: significance level.

## Data Availability

The article data are available in the different tables and figures of the article or upon request to the corresponding author.
